# Gastric Leiomyosarcoma: A Case Report and Literature Review

**DOI:** 10.7759/cureus.94111

**Published:** 2025-10-08

**Authors:** João Mendes, Fábio Viveiros, Manuel Veiga, Francisco Fazeres, Eduardo Vasconcelos

**Affiliations:** 1 General Surgery, Unidade Local de Saúde (ULS) do Alto Minho, Viana do Castelo, PRT; 2 Pathology, Unidade Local de Saúde (ULS) do Alto Minho, Viana do Castelo, PRT

**Keywords:** cancer, case report, gastrointestinal stromal tumor (gist), leiomyosarcoma, malignant, stomach

## Abstract

Gastric leiomyosarcoma is an exceptionally rare malignancy. This case report describes a 57-year-old woman, previously healthy, who presented to the emergency department with signs of upper gastrointestinal bleeding and severe anemia. Urgent upper endoscopy revealed a large ulcerated lesion on the greater curvature of the stomach, raising suspicion for a gastrointestinal stromal tumor (GIST). Biopsies suggested a mesenchymal neoplasm with epithelioid features. Staging imaging excluded metastatic disease. The patient underwent a laparoscopic radical subtotal gastrectomy with Roux-en-Y reconstruction. Histological and immunohistochemical analysis concluded a diagnosis of gastric leimyosarcoma. Surgical margins were negative, and no lymph node metastases were found. This case underscores the importance of immunohistochemistry in differentiating leiomyosarcoma from other mesenchymal tumors such as GIST. Despite surgical resection being the mainstay of treatment, the prognosis remains guarded due to high mitotic activity and the potential for recurrence. This report adds to the limited literature and highlights the need for further studies to establish standardized management protocols.

## Introduction

Leiomyosarcomas are malignant tumors originating from smooth muscle cells, frequently found in the uterus and retroperitoneum, but very rarely in the stomach. Gastric leiomyosarcomas account for less than 1% of all gastric malignancies and must be distinguished from gastrointestinal stromal tumors (GISTs), which are significantly more prevalent and exhibit different biological behaviors and responses to treatment [1,2]. Since the early 2000s, fewer than two dozen cases have been reported in the English literature, underscoring the exceptional rarity of this tumor entity [3]. Histologically, leiomyosarcomas are composed of spindle cells with abundant eosinophilic cytoplasm, nuclear pleomorphism, and a high mitotic index [4,5]. The diagnosis relies on immunohistochemical staining, typically positive for smooth muscle markers such as alpha-smooth muscle actin (α-SMA) and desmin, and negative for CD117 (c-KIT), DOG1, and CD34, which are more characteristic of GISTs [6,7].

The discovery of c-KIT (CD117) and its overexpression in GISTs in the late 1990s revolutionized the diagnostic landscape of mesenchymal tumors of the gastrointestinal tract. This marker not only enabled the clear distinction between GISTs and true smooth muscle tumors like leiomyosarcomas, but also opened the door for targeted therapy with tyrosine kinase inhibitors such as imatinib [8]. However, a small subset of GISTs are c-KIT negative, creating diagnostic overlap. In this context, DOG1 emerged as a sensitive and specific marker, especially useful in c-KIT-negative GISTs [8]. These immunohistochemical advances have made it possible to more accurately classify gastric mesenchymal tumors and guide treatment decisions appropriately.

Because of its rarity, gastric leiomyosarcoma poses a diagnostic challenge and lacks consensus on optimal management strategies, although complete surgical excision remains the treatment of choice [5,9]. This report aims to contribute to the growing but still limited literature on this rare malignancy.

## Case presentation

**Figure 1:**
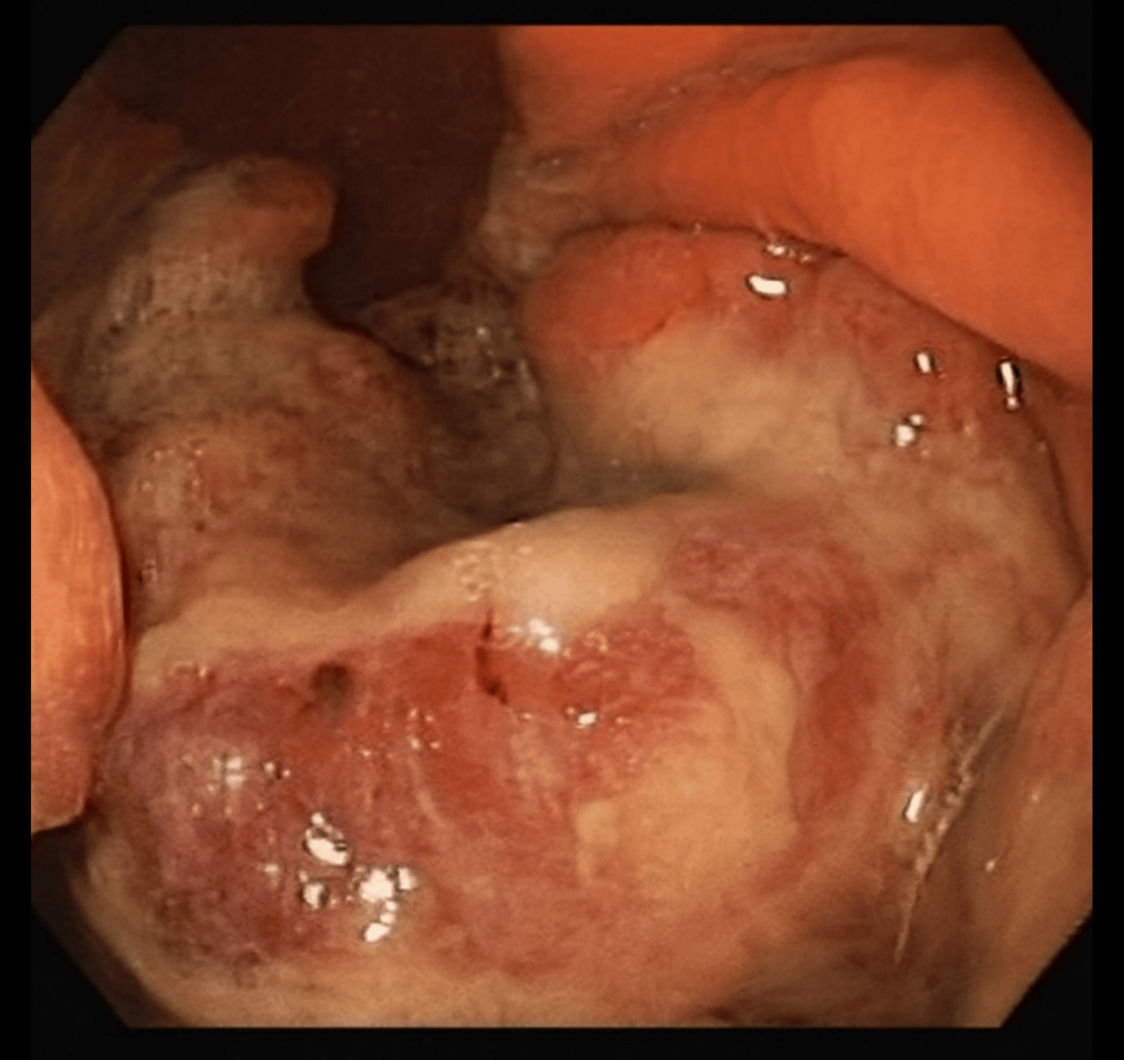
Ulcerated mass in the body-antrum transition.

A 57-year-old woman with no significant past medical history presented in December 2024 with dark stools consistent with melena, evolving for one week. She was referred to the emergency department following an episode of syncope, along with complaints of pallor and headaches. Laboratory tests revealed a hemoglobin level of 5.5 g/dL (reference range: 12-16 g/dL), microcytic and hypochromic. The remaining metabolic profile showed no significant abnormalities. She underwent urgent upper endoscopy, which identified a large ulcerated lesion on the greater curvature at the body-antrum transition (Figure 1). The lesion had irregular borders and caused regional deformity, but showed no active bleeding. 

Endoscopic biopsies suggested a mesenchymal tumor resembling epithelioid GIST. Histologically, the neoplastic cells displayed an epithelioid morphology arranged in solid and trabecular patterns, with abundant eosinophilic cytoplasm and focal vacuolization. Initial immunohistochemical analysis (Table 1) revealed multifocal positivity for HepPar and a high proliferative index with Ki-67 exceeding 80%, while markers such as CK20, CK7, CDX2, and chromogranin were negative, raising suspicion for a poorly differentiated neoplasm with hepatocellular features. However, extended immunohistochemical studies (Table 1) later demonstrated diffuse positivity for DOG1 and smooth muscle actin (SMA), with negative staining for CD117, CD34, S100, and desmin, raising suspicion for a leiomyosarcoma-type gastric stromal tumor. 

**Table 1:** Immunohistochemical panel results for gastric tumor biopsy This table includes both the initial immunohistochemical findings and the additional findings performed for further characterization.

Marker	Result
HepPar	Positive (multifocal)
CK20	Negative
CK7	Negative
CDX2	Negative
Chromogranin	Negative
Ki-67	High (>80%)
DOG1	Positive
Actin	Positive
Desmin	Negative
CD34	Negative
CD117	Negative
S100	Negative
HMB45	Negative
CK8/18	Negative
CD45	Negative
Glypican	Negative
CD30	Negative
CK AE1/AE3	Negative
Alpha Fetoprotein (AFP)	Negative
CDX2 (2nd time)	Negative
CK7 (2nd time)	Negative
Chromogranin (2nd time)	Negative
HepPar 1	Positive (likely nonspecific)
HSP70	Positive (likely nonspecific)

**Figure 2:**
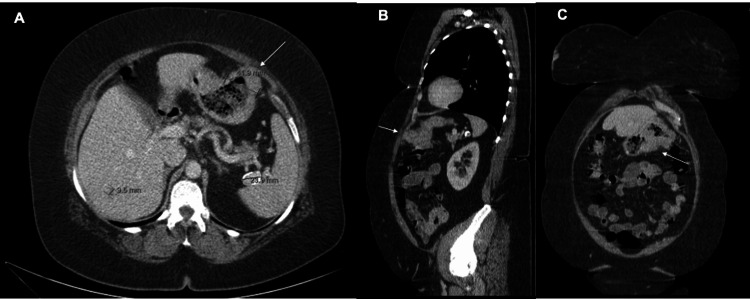
Contrast-enhanced abdominal CT scan showing the gastric lesion. (A) Axial view revealing an exophytic gastric mass measuring approximately 41.9 mm originating from the greater curvature, with irregular borders and surrounding fat stranding (white arrow). A concurrent 9.5 mm hepatic lesion is also identified, consistent with a hemangioma. (B) Sagittal reconstruction demonstrating the mass projecting anteriorly from the gastric wall. (C) Coronal view confirming the extraluminal growth pattern and spatial relationship with adjacent structures (white arrow).

Staging studies, including computed tomography (CT) of the thorax-abdomen-pelvis and abdominal magnetic resonance imaging (MRI), identified a 42 mm exophytic gastric mass with peripheral fat stranding and a 6 mm perilesional lymph node (Figure 2). A concurrent hepatic lesion was consistent with a benign hemangioma.

Following multidisciplinary oncologic discussion, radical surgical resection was recommended, as melena persisted intermittently during the hospitalization despite supportive care. The patient underwent laparoscopic subtotal radical gastrectomy with Roux-en-Y reconstruction. No major postoperative complications occurred. 

**Figure 3:**
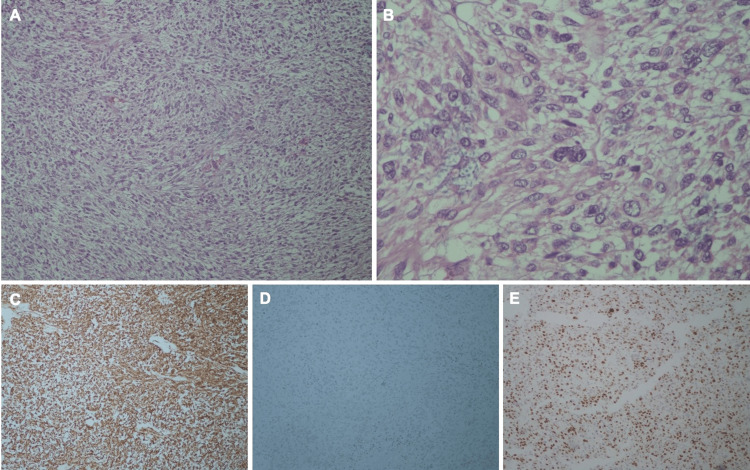
Histological and immunohistochemical features of the tumor. (A) Hematoxylin and eosin (H&E) staining, 10× magnification: neoplastic proliferation composed of spindle to oval-shaped cells with a stromal pattern. (B) H&E staining, 40× magnification: cellular detail showing marked nuclear atypia. (C) Immunohistochemistry (IHC) for α-smooth muscle actin (α-SMA): diffuse and strong cytoplasmic positivity. (D) IHC for CD117 (C-KIT): negative staining. (E) IHC for Ki-67: high proliferative index with approximately 75% of cells showing nuclear positivity.

Final pathology examination (Figure 3) revealed a 10 x 10 cm mass infiltrating the gastric wall to the subserosa, with extensive necrosis and numerous pleomorphic spindle and oval cells. A total of 260 mitoses per 50 high-power fields were observed. Immunohistochemistry showed diffuse positivity for α-SMA, negativity for CD117 and CD34, and a Ki-67 index of 75%. All surgical margins were free of tumor, and none of the 13 regional lymph nodes showed metastatic involvement.

The patient was subsequently referred to a specialized oncology center for pathological review, which confirmed a high-grade spindle cell neoplasm with marked pleomorphism, high mitotic index, hemorrhage and necrosis, diffuse positivity for h-caldesmon and α-SMA, and negativity for CD117 and DOG1. The most likely diagnosis was considered a grade 3 leiomyosarcoma. This highlights the diagnostic complexity of such tumors and the critical role of comprehensive immunohistochemical profiling in establishing a definitive diagnosis.

Approximately three months postoperatively, a positron emission tomography (PET)-CT scan revealed local recurrence, multiple hepatic metastases, and peritoneal carcinomatosis. The patient was proposed for palliative systemic chemotherapy, but unfortunately passed away seven months postoperatively due to disease progression.

## Discussion

Due to its rarity, gastric leiomyosarcoma is often misdiagnosed as GIST, which can delay appropriate management and impact patient outcomes. Gastric leiomyosarcomas account for approximately 10% of clinically relevant gastrointestinal mesenchymal tumors and share many histological features with GISTs, making differentiation challenging. Histologically, they are typically composed of spindle cells with variable pleomorphism, whereas GISTs may show spindle, epithelioid, or mixed morphology. Immunohistochemistry remains the cornerstone for accurate diagnosis, as leiomyosarcomas are almost always diffusely positive for α-SMA and variably positive for desmin, but typically negative for CD117 (c-KIT) and DOG1, which are key markers for diagnosing GISTs [6,7,10,11]. CD34 is typically positive in gastric GISTs, as it is present in over 90% of cases, its absence can help in distinguishing other mesenchymal tumors. CD34 may be positive in some leiomyosarcomas, but this is not a consistent feature, whereas most gastric GISTs are strongly positive for CD117, DOG1, and CD34, and variably express α-SMA [11]. The expression of h-caldesmon, a muscle-specific actin-binding protein, provides additional evidence of smooth muscle differentiation and helps distinguish leiomyosarcomas from GISTs [4].

In our case, the initial endoscopic findings raised suspicion for a GIST, given the presence of a large ulcerated gastric lesion. Histologically, the tumor was predominantly composed of spindle cells, which further supported a mesenchymal neoplasm. The initial biopsy immunohistochemistry showed DOG1 positivity, raising the possibility of GIST; however, the final surgical specimen demonstrated a classic leiomyosarcoma immunoprofile. Specifically, the tumor was negative for CD117, DOG1, and CD34, diffusely positive for α-SMA and h-caldesmon, and exhibited a high mitotic index. The initial DOG1 positivity likely reflected the presence of interstitial cells of Cajal (ICCs) within the biopsy rather than true tumor expression [11]. These findings highlight the critical role of comprehensive immunohistochemical analysis and the potential pitfalls of relying solely on biopsy specimens for diagnosis. The combination of spindle cell morphology, α-SMA and h-caldesmon positivity, CD117 and DOG1 negativity, and high mitotic activity is consistent with the typical profile of gastric leiomyosarcomas and allows differentiation from GISTs, which usually express CD117, DOG1, and frequently CD34 [6,7,10,11].

Radical surgical resection with negative margins is considered the gold standard for treatment [1,9]. In contrast to GIST, where targeted therapy with tyrosine kinase inhibitors like imatinib is effective, leiomyosarcomas are relatively chemoresistant and show limited response to radiotherapy [4,12]. Prognostic factors include tumor size, mitotic rate, and completeness of surgical resection. In a review of similar cases, tumors larger than 5 cm with high mitotic activity had a poor prognosis, with increased risk of recurrence and metastasis [5,10,13]. The classification by Miettinen and Lasota (Table 2) provides a risk stratification system based on these factors [14], with our case corresponding to a high risk of malignant progression. This highlights the importance of early and accurate diagnosis to optimize surgical planning.

**Table 2:** Risk stratification of GIST based on mitotic rate, tumor size, and anatomical site. This classification, proposed by Miettinen and Lasota [14], categorizes GISTs according to their malignant potential using three key parameters: mitotic count per 50 high-power fields (HPF), tumor size, and anatomical location (stomach, duodenum, jejunum/ileum, or rectum). Risk levels range from none to high, with higher mitotic rates and larger tumor sizes generally associated with increased malignant potential. Sites such as the small intestine and rectum demonstrate a higher risk for equivalent size and mitotic index compared to gastric tumors. Data are limited for some subgroups (e.g., small tumors with high mitotic activity). Table Source: Used with permission of Nancy International Ltd Subsidiary AME Publishing Company, from Gastrointestinal Stromal Tumor, Zhao and Yue, 2012 [8]; permission conveyed through Copyright Clearance Center, Inc.

Mitotic rate (50 HPF)	Tumor size (cm)	Stomach	Duodenum	Jejunum or ileum	Rectum
5	2	None	None	None	None
	>2 - 5	Very low	Low	Low	Low
	>5 - 10	Low	Moderate	Insufficient data	Insufficient data
	>10	Moderate	High	High	High
>5	2	None	High	Insufficient data	High
	>2 - 5	Moderate	High	High	High
	>5 - 10	High	High	Insufficient data	Insufficient data
	>10	High	High	High	High

In addition, the Grupo Español de Investigación en Sarcomas (GEIS) guidelines for gastrointestinal sarcomas stress the importance of accurate histologic subtyping for clinical decision-making and emphasize that surgery is the only potentially curative option for non-GIST sarcomas [15]. Our findings align with previously reported cases, such as those by Gubatan et al. [3], Kang et al. [4], and Hasnaoui et al. [5], who also documented large, exophytic lesions with high mitotic indices and challenging diagnostic pathways.

This case contributes to the scarce body of evidence on gastric leiomyosarcoma, highlighting the diagnostic complexity, the relevance of modern immunohistochemistry, and the need for surgical expertise. While the clinical course was favorable in the immediate postoperative period, long-term surveillance remains crucial due to the elevated risk of recurrence and limited options for adjuvant therapy.

## Conclusions

Gastric leiomyosarcoma is a rare and aggressive tumor that often presents diagnostic difficulties due to its similarity to other mesenchymal neoplasms. A comprehensive histopathological and immunohistochemical evaluation is crucial to distinguish it from more common entities like GIST. The absence of CD117 and DOG1 and the presence of smooth muscle markers such as α-SMA can aid in the diagnosis.

Surgical treatment remains the mainstay of treatment and provides the best opportunity for long-term survival. Given the high mitotic index and potential for recurrence, careful postoperative monitoring is advised. Additional case reports and pooled analyses are needed to establish clearer guidelines for management and prognosis.

## References

[1] Kang WZ, Xue LY, Tian YT (2019). Leiomyosarcoma of the stomach: a case report. World J Clin Cases.

[2] Wang T, Zreik R, Leng B (2023). Primary gastric leiomyosarcoma: a rare case. Cureus.

[3] Gubatan J, Shah N (2020). Gastric leiomyosarcoma unmasked by bleeding from a percutaneous endoscopic gastrostomy tube. ACG Case Rep J.

[4] Garg R, AlRajjal A, Berri R, Barawi M (2020). Primary gastric leiomyosarcoma: a case report and review of the literature. J Gastrointest Cancer.

[5] Hasnaoui A, Jouini R, Haddad D, Zaafouri H, Bouhafa A, Ben Maamer A, Ben Brahim E (2018). Gastric leiomyosarcoma and diagnostic pitfalls: a case report. BMC Surg.

[6] Al-Yousofy F, Alshargabi G, Ahmed F, Almohtadi A, Fazea M, Altam A (2022). Primary huge gastric leiomyosarcoma with multiple metastases in a 60-year-old female: a case report. Pan Afr Med J.

[7] Kitagawa H, Kaneko M, Kano M (2018). Coexistence of gastrointestinal stromal tumor and leiomyosarcoma of the stomach presenting as a collision tumor: a case report and review of literature. Pathol Int.

[8] Zhao X, Yue C (2012). Gastrointestinal stromal tumor. J Gastrointest Oncol.

[9] Sequeira C, Coelho M, Santos I, Lopes S, Fonseca R, Mangualde J, Oliveira AP (2023). Primary gastric leiomyosarcoma presenting as a giant polyp. Rev Esp Enferm Dig.

[10] Cheng CS, Chen L, Xie J, Chen Z (2019). Multimodality palliative treatment with transarterial chemoembolization and high-intensity focused ultrasound for gastric leiomyosarcoma multiple liver metastasis pain: a case report. Medicine (Baltimore).

[11] Hirota S (2018). Differential diagnosis of gastrointestinal stromal tumor by histopathology and immunohistochemistry. Transl Gastroenterol Hepatol.

[12] Yamamoto A, Tateishi Y, Aikou S, Seto Y, Ushiku T (2021). The first case of gastric leiomyosarcoma developed through malignant transformation of leiomyoma. Pathol Int.

[13] Ahsan N, Sharma M (2024). From rarity to revelation: shedding light on gastric leiomyosarcoma in a small needle core biopsy - a case report. Int J Med Sci Innov Res.

[14] Miettinen M, Lasota J (2006). Gastrointestinal stromal tumors: pathology and prognosis at different sites. Semin Diagn Pathol.

[15] Poveda A, García Del Muro X, López-Guerrero JA (2017). GEIS guidelines for gastrointestinal sarcomas (GIST). Cancer Treat Rev.

